# Evaluating Genome Assemblies for Optimized Completeness and Accuracy of Reference Gene Sequences in Wheat, Rye, and Triticale

**DOI:** 10.3390/plants14071140

**Published:** 2025-04-06

**Authors:** Mingke Yan, Guodong Yang, Dongming Yang, Xin Zhang, Quanzhen Wang, Jinghui Gao, Chugang Mei

**Affiliations:** 1 College of Grassland Agriculture, Northwest A&F University, Yangling 712100, China; 2 College of Plant Protection, Northwest A&F University, Yangling 712100, China

**Keywords:** genome, gene sequence completeness, quantitative gene expression, BUSCO, RNA-seq, wheat, rye, triticale

## Abstract

Recent years have witnessed a surge in the publication of dozens of genome assemblies for Triticeae crops, which have significantly advanced gene-related research in wheat, rye, and triticale. However, this progress has also introduced challenges in selecting universally efficient and applicable reference genomes for genotypes with distant or ambiguous phylogenetic relationships. In this study, we assessed the completeness and accuracy of genome assemblies for wheat, rye, and triticale using comparative benchmarking universal single-copy orthologue (BUSCO) analysis and transcript mapping approaches. BUSCO analysis revealed that the proportion of complete genes positively correlated with RNA-seq read mappability, while the frequency of internal stop codons served as a significant negative indicator of assembly accuracy and RNA-seq data mappability in wheat. By integrated analysis of alignment rate, covered length, and total depth from RNA-seq data, we identified the assemblies of SY Mattis, Lo7, and SY Mattis plus Lo7 as the most robust references for gene-related studies in wheat, rye, and triticale, respectively. Furthermore, we recommend that the D genome sequence be incorporated in reference assemblies in bioinformatic analyses for triticale, as introgression, translocation, and substitution of the D genome into triticale genome frequently occurs during triticale breeding. The frequency of internal stop codons could help in evaluating correctness of assemblies published in the future, and other findings are expected to support gene-related research in wheat, rye, triticale, and other closely related species.

## 1. Introduction

Wheat (*Triticum aestivum* L.), rye (*Secale cereale* L.), and triticale (× *Triticosecale* Wittmack) are members of the wheat tribe (Triticeae) within the Pooideae subfamily of grasses. Wheat is one of the most widely grown crops used as eaten cereals throughout the world. Rye and triticale can also serve human culinary uses, but are more often cultivated as cover crops and important forage grasses. These Triticeae species contain the basic chromosome set with a number of 7, and the entire genomes are often very complex and large (e.g., diploid rye, ~7–8 Gb; tetraploid wheat, >10 Gb; hexaploid wheat, ~16 Gb; hexaploid triticale, ~17 Gb), and more than 80% of each genome is composed of repetitive DNA sequences [1,2,3,4].

A new member of the Triticeae species, triticale is a synthetic hybrid derived by crossing wheat (*T. durum* L., AABB, or *T. aestivum* L., AABBDD) and rye (*S. cereale* L., RR). As a result, triticale may be hexaploid (2N = 42; AABBRR) or octoploid (2N = 56; AABBDDRR) depending on which category of wheat is crossed with rye [5]. Most commonly grown triticale are hexaploids, because the large genome of octoploid types renders them less stable. Other ploidy levels such as decaploid and tetraploid exist, but are rarer [5]. After a breeding history of nearly 150 years, most modern triticale cultivars are derived from crossing two triticale parents or from crossing wheat with triticale [5,6]. As the D subgenome (~3.95 Gb) is smaller than the R subgenome (~7–8 Gb), the triticale genome may be more complicated than common wheat [7,8].

In efforts to accelerate the breeding process, recent technological advances in high-throughput DNA sequencing, high-throughput chromosome conformation capture (Hi-C), optical map-assisted assembly, and data analysis algorithms have resulted in significant progress in the genome sequencing of Triticeae crops [7,9]. However, hampered by the highly complex structure, assembled chromosome-scale genomes of polyploid wheat were not released until 2017 [10,11]. Since then, dozens of high-quality wheat and rye genome assemblies have been released (a subset of chromosome-scale assemblies, which were used as references in the current study, are listed in Table 1). Coupled with the genome sequences, tens of thousands to hundreds of thousands of genes have been predicted or annotated (34,441–43,928 in rye; 66,559–88,002 in tetraploid wheat; 105,692–147,646 in hexaploid wheat) (Table 1). These resources have greatly accelerated the identification and valuation of elite genes/alleles and their application in improvements in wheat and rye and their derivative triticale.

One of the main objectives of sequencing a genome is to obtain its catalogue of genes. The gene space (i.e., the part of the genome comprising all the genes and gene-related DNA) in a genome is the core focus of genetic, transcriptomic, proteomic, and evolutionary studies. Denton et al. [12] compared multiple draft assemblies against higher-quality versions and discovered that over 40% of all gene families are estimated to have an inaccurate number of genes in draft assemblies and that these incorrect assemblies both add and subtract genes. In addition, the large numbers of fragmented genes in low-quality genomes can significantly impede efficient genetic research [12]. Moreover, reference-based transcriptomic studies have been reported to be more convenient, productive, and accurate in most cases [13]. RNA-seq-based gene expression quantification achieves high accuracy when the reference genome and query samples share identical genotypes. However, cross-mapping reads to genomes of different genotypes introduces measurable reference bias correlating with genetic distance [14]. Since genomic data have become available, the assembly of the bread wheat cultivar Chinese spring has been widely used as reference for wheat and triticale (e.g., [1,15,16,17,18]) or Lo7 assembly in rye studies [19,20,21]. When more genome assemblies become available, whether such choices are still optimal should be reexamined. More appropriate reference data would help in assessing gene expression and structure and in detecting new genetic information more comprehensively and accurately. Therefore, given the challenges associated with processing large-scale genomic datasets in downstream research, evaluating existing assemblies to select the most complete, accurate, and robust reference data with wide applicability is of high value.

The completeness and correctness of gene sequences serve as critical quality indicators for genome assemblies [22,23,24]. To evaluate the completeness of gene space, also known as functional completeness, a widely used metric is Benchmarking Universal Single-Copy Orthologs (BUSCO). BUSCO evaluates the presence of a predefined set of highly conserved orthologous genes as a proxy for genome-wide completeness [25]. However, BUSCO may be ineffective for gauging assembly completeness in certain species or situations, such as in genes with long introns [22]. Another metric for functional completeness is transcript mappability [24,26]. Inadequate coverage can lead to assembly defects, such as premature stop codons and frame-shift indel errors. These and other assembly errors critically affect transcript mapping, resulting in downstream errors that obscure meaningful signals and propagate into false candidate gene identification and inaccurate gene annotation [24,27]. These assembly defects fall under the issues of assembly correctness, which is challenging to assess directly [22]. Therefore, comparing the functional completeness and correctness of different genome assemblies can facilitate transcriptomic and gene-related studies.

To evaluate the mapping performance of RNA-seq data, three frequently used metrics are overall alignment rate, coverage, and depth. Alignment rate (or mapping rate) reflects read-level mapping efficiency, while coverage and depth represent base-level mapping statistics [28]. Since coverage is a proportion or percentage, the corresponding absolute value of covered length or total covered sites may be a better metric to evaluate mappability across different genome assemblies. Additionally, the accuracy of RNA-seq improves with increasing sequencing depth, and higher depth is generally preferred for correctly determining transcript and base abundance [29,30,31].

In order to facilitate future gene-related studies in wheat, rye and triticale, in this study, we performed a comprehensive evaluation of functional completeness and correctness of publicly available genomic references using BUSCO analysis and RNA-seq read mapping methodologies. Three groups of assemblies were proposed as high-confidence references with wide applicability for gene-related studies in wheat, rye, and triticale, respectively. Based on the evaluation, new metrics were proposed for future genome assembly. Our findings will assist future transcriptomic, genetic, proteomic, and evolutionary studies in the three crops.

## 2. Results

### 2.1. Differences in the Key Parameters of Triticeae Genome Assemblies

A total of 41 chromosome-scale genome assemblies were compared in this study, including 37 allohexaploid wheat (AABBDD), 2 allotetraploid wheat (AABB) and 2 rye (RR) genomes (Table 1). The size of the *T. aestivum* assemblies varied between 14.29 Gb and 15.10 Gb. The assembly sizes of the two allotetraploid wheat genomes were very close (10.46 Gb and 10.68 Gb), while the two rye assemblies differed greatly (6.74 Gb and 7.74 Gb). The number of predicted genes was overall in concordance with the assembly size: 105,692–147,646 in allohexaploid wheat, 66,559–88,002 in allotetraploid wheat, and 34,441–43,928 in rye. Assembly continuity metrics revealed that N50 values of contigs varied dramatically among the assemblies, ranging from 56 Kb in Svevo to 58 Mb in Chuanmai104. In comparison, N50 values of scaffolds across assemblies were closer, especially in wheat (smallest: 688 Mb in LongReach_Lancer; largest: 747 Mb in Zavitan ).

**Table 1:** Statistics of the Triticeae assemblies compared in this study.

Species	Genotype	Genome Type	Assembly Length (Gb)	Predicted Gene Number *	Number of Contigs	Number of Scaffolds	N50 of Contigs	N50 of Scaffolds	Percent of Gaps	Assembly Version	Reference
*Triticum aestivum*	Chinese Spring	AABBDD	14.58	106,914	306,707	22	341 Kb	713 Mb	1.78%	v2.1	[32]
	Zang1817	AABBDD	14.71	107,336	822,893	383,261	66 Kb	704 Mb	1.05%	v1	[33]
	ArinaLrFor	AABBDD	14.66	117,569	501,458	22	80 Kb	697 Mb	0.82%	v3.0	[3]
	Jagger	AABBDD	14.55	115,873	705,558	22	85 Kb	705 Mb	1.67%	v1.1	
	Julius	AABBDD	14.39	115,552	620,780	22	77 Kb	708 Mb	1.21%	v1.0	
	Landmark	AABBDD	14.44	115,515	662,766	22	81 Kb	705 Mb	1.92%	v1.0	
	LongReach Lancer	AABBDD	14.29	116,473	508,146	22	75 Kb	688 Mb	0.81%	v1.0	
	Mace	AABBDD	14.36	116,367	533,095	22	71 Kb	690 Mb	0.87%	v1.0	
	Norin61	AABBDD	14.91	116,106	462,063	22	66 Kb	712 Mb	0.78%	v1.1	
	spelta_PI190962	AABBDD	14.45	116,395	487,212	22	83 Kb	707 Mb	0.76%	v1.0	
	Stanley	AABBDD	14.47	115,781	603,408	22	71 Kb	713 Mb	1.68%	v1.2	
	SY Mattis	AABBDD	14.96	117,276	1,063,653	22	76 Kb	698 Mb	1.03%	v1.0	
	Fielder	AABBDD	14.70	116,480	5202	3795	20 Mb	717 Mb	0.00%	v1	[34]
	Kariega	AABBDD	14.68	116,838	7395	22	26 Mb	717 Mb	0.13%	v1	[35]
	Renan	AABBDD	14.26	109,653	12,779	359	2 Mb	703 Mb	1.79%	v2.1	[36]
	Attraktion	AABBDD	14.24	NA	1553	21	17 Mb	703 Mb	0.00%	NA	[37]
	Kenong9204	AABBDD	14.47	105,692	102,806	22	373 Kb	714 Mb	0.74%	NA	[38]
	Aikang58	AABBDD	14.76	119,350	279,897	158,176	237 Kb	717 Mb	0.63%	NA	[39]
	Chuanmai104	AABBDD	14.78	122,554	6065	5398	58 Mb	727 Mb	0.00%	v2.0	[40]
	CWI86942	AABBDD	14.57	147,646	4780	22	44 Mb	726 Mb	0.00%	v1	[41]
	Abbondanza	AABBDD	14.70	134,090	7905	6567	25 Mb	719 Mb	0.00%	NA	[42]
	Aimengniu	AABBDD	15.01	135,639	19,536	18,163	24 Mb	727 Mb	0.00%	NA	
	Beijing8	AABBDD	14.83	136,169	5995	3708	19 Mb	718 Mb	0.00%	NA	
	Chuanmai42	AABBDD	14.66	133,391	4876	3906	41 Mb	722 Mb	0.00%	NA	
	Handan6172	AABBDD	15.10	138,709	11,413	8862	26 Mb	735 Mb	0.00%	NA	
	Jimai22	AABBDD	14.62	134,424	5417	4268	33 Mb	726 Mb	0.00%	NA	
	Jinmai47	AABBDD	15.05	136,282	8175	2790	21 Mb	744 Mb	0.00%	NA	
	Kuofan11	AABBDD	14.86	133,789	9446	8185	30 Mb	726 Mb	0.00%	NA	
	Mazhamai	AABBDD	14.67	135,896	6584	4985	32 Mb	721 Mb	0.00%	NA	
	Ningchun4	AABBDD	14.58	133,773	4996	3649	31 Mb	714 Mb	0.00%	NA	
	Shi4185	AABBDD	14.99	134,130	11,963	9171	26 Mb	738 Mb	0.00%	NA	
	Xiaoyan6	AABBDD	14.87	138,457	14,981	13,674	25 Mb	720 Mb	0.00%	NA	
	Xinong6028	AABBDD	14.69	136,471	4827	3522	31 Mb	723 Mb	0.00%	NA	
	Yangmai158	AABBDD	14.70	135,207	6028	4033	25 Mb	714 Mb	0.00%	NA	
	Zhengmai366	AABBDD	14.83	135,296	11,915	8003	26 Mb	735 Mb	0.00%	NA	
	Zhoumai16	AABBDD	15.06	136,271	6413	3366	33 Mb	742 Mb	0.00%	NA	
	Zhoumai22	AABBDD	15.04	135,054	13,808	10,954	26 Mb	729 Mb	0.00%	NA	
*Triticum turgidum*	Svevo	AABB	10.46	66,559	476,825	15	56 Kb	722 Mb	1.55%	v1	[4]
	Zavitan	AABB	10.68	88,002	492,885	148,296	57 Kb	747 Mb	3.31%	v2.1	[43]
*Secale cereale*	Lo7	RR	6.74	34,441	314,632	8	72 Kb	900 Mb	2.31%	v1p1p1	[44]
	Weining	RR	7.74	43,928	71,705	8	441 Kb	1024 Mb	0.10%	NA	[8]

* Limited to high-confidence genes (if available). NA, not available.

**Figure 1:**
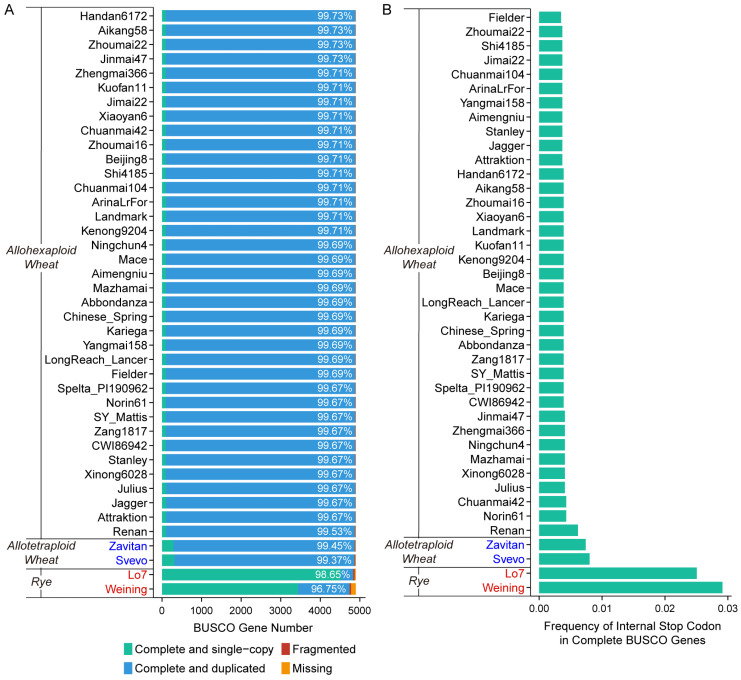
BUSCO analysis of wheat, rye, and triticale assemblies. (**A**) Distribution of different categories of BUSCO genes. The percentages of complete BUSCO genes (both single-copy and duplicated complete genes) for each line were marked on corresponding bars. (**B**) Frequency of internal stop codon inside the total complete BUSCO genes.

Gene completeness assessment by BUSCO evaluation revealed that all the wheat assemblies showed high completeness, as the complete BUSCO gene percentages were estimated to be over 99.37%. By contrast, the diploid rye exhibited slightly lower complete BUSCO gene percentages: 98.65% for Lo7 and 96.75% for Weining (Figure 1A). Furthermore, we identified a differentially distributed frequency of internal stop codons in BUSCO genes among genotypes, which was overall reversely correlated with the complete BUSCO gene percentage (Figure 1B). The frequency of internal stop codons served as an indicator for deciphering gene mapping efficiency and will be further addressed in later sections.

### 2.2. Efficiency Differences in Reference-Based Transcript Mapping in Wheat

**Figure 2:**
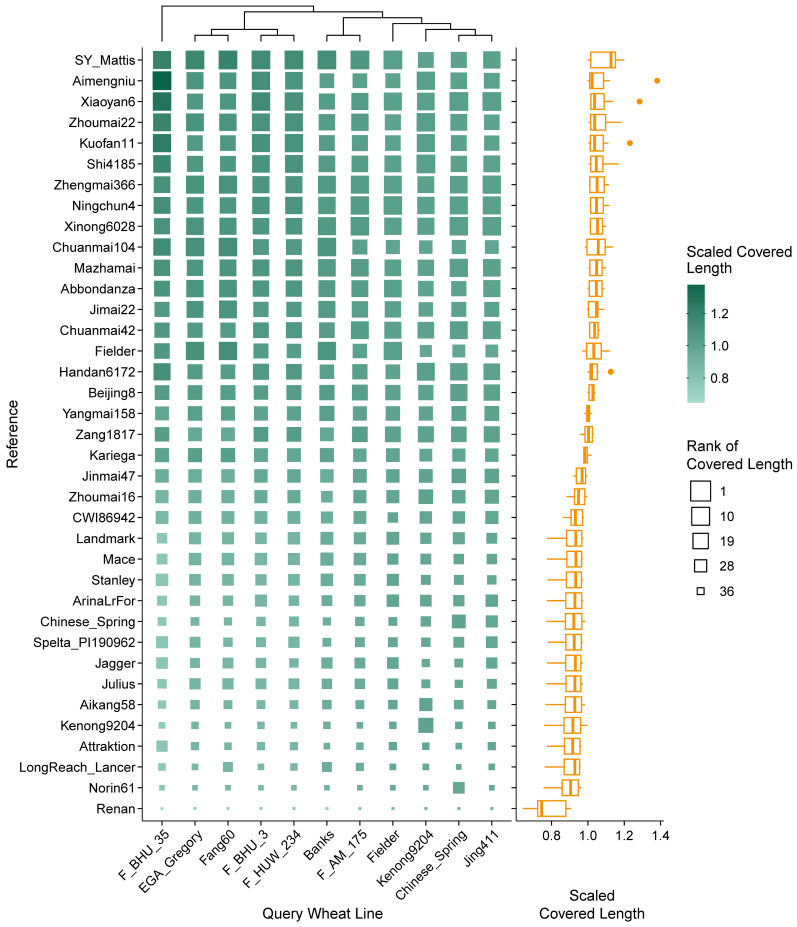
Covered length distribution of RNA-seq reads derived from different wheat varieties mapped to different wheat genome references. Scaled covered lengths are displayed via heatmap on the left and corresponding boxplot (statistics based on each reference) on the right. Original covered lengths are scaled according to each query wheat line and ranked in descending order (e.g., F_BHU_35 received the highest and lowest covered length when mapped to Aimengniu and Renan, respectively). The references are sorted according to the average scaled covered length of all varieties.

**Figure 3:**
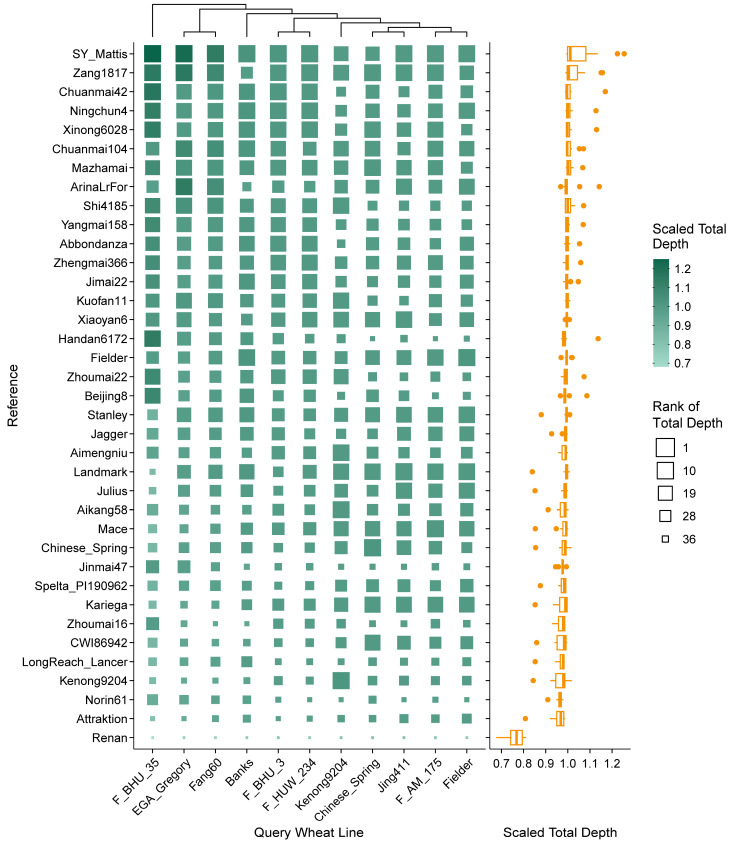
Total depth distribution of RNA-seq reads derived from different wheat varieties mapped to different wheat genome references. Scaled total depths are displayed via heatmap on the left and corresponding boxplot (statistics based on each reference) on the right. Original total depths are scaled according to each query wheat line and ranked in descending order (e.g., F_BHU_35 received the highest and lowest total depth when mapped to SY_Mattis and Renan, respectively). The references are sorted according to the average scaled total depth of all varieties.

To evaluate the fitness and efficiency of different reference genome assemblies in gene study in wheat, rye and triticale, cDNA sequences represented by RNA-seq reads of the crop lines originating from different regions of the world (Table S1) were mapped to different genome assemblies. Mapping results of 11 wheat lines revealed that the alignment rate ranged from 74.6% (EGA_Gregory mapped to Renan) to 98.6% (Fielder mapped to Fielder). The reference-wise distribution of read coverage and depth was in a similar pattern irrespective of query lines, while the variation of alignment rate was more inconsistent. On average, using SY Mattis as reference achieved high alignment rate, covered length, and total depth. Other references with high alignment rate included Zang1817, Beijing8, Xiaoyan6, and Shi4185 (Figure S1); references with high covered length included Aimengniu, Xiaoyan6, Zhoumai22, and Kuofan11 (Figure 2); references with high total depth included Zang1817, Chuanmai42, Ningchun4, and Xinong6028 (Figure 3). In contrast, Renan, Attraktion, LongReach_Lancer, and Kenong9204 were the poorly mapped references in all aspects. Taking query line Jing411 as an example, the difference between the highest (95.1%) and lowest (90.5%) alignment rates corresponded to a gap of 5,369,097 read pairs; the difference between the highest and lowest covered lengths was 35,397,977 bp (0.24% of the Chinese Spring assembly; 15.78% of the total length of Chinese Spring HC exons); the difference between the highest and lowest total depths was 5,931,922,239 bp (0.41× of the Chinese Spring assembly; 26.44× of the total length of Chinese Spring HC exons). It should be noted that RNA-seq reads mapped to assemblies of the identical genotypic source always performed well in all indices, as proved in Chinese Spring, Fielder, and Kenong9204.

**Figure 4:**
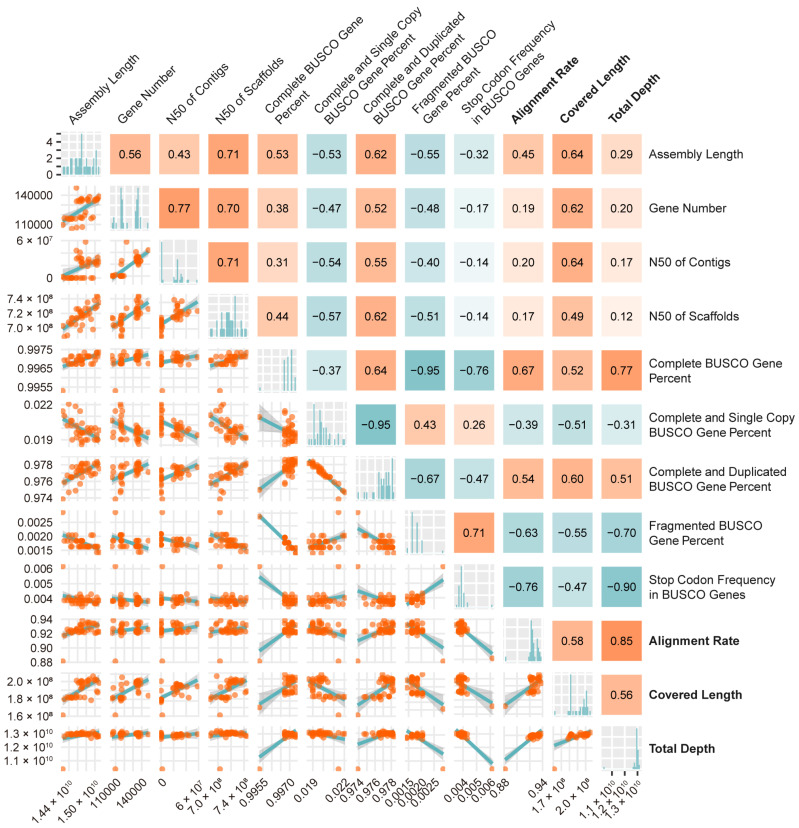
Correlation analysis between reference assembly information and wheat RNA-seq read mapping statistics. The lower triangular matrix is composed of the bivariate scatter plots with a fitted smooth line. The upper triangular matrix shows the values and heatmap of Pearson correlation. The colors of the heatmap were assigned according to the correlation coefficient, as indicated by the color scale. The diagonal plots represent histogram distributions for each measurement. The average values of alignment rate, covered length and total depth obtained for each reference over different query wheat lines were employed in correlation analysis. Genome statistics of ‘Attraktion’ were excluded from correlation analysis due to lack of annotation data.

To explore assembly indicators putatively determining or reflecting alignment efficiency, a correlation analysis was performed between the major reference assembly quality indices and wheat cDNA mapping results. This revealed a high negative correlation between stop codon frequency in BUSCO genes and alignment depth, and the former was also moderately negatively correlated with alignment rate. In addition, all the alignment indices were moderately correlated with fragmented BUSCO gene percentage (negative), complete BUSCO gene percentage (positive), and complete and duplicated BUSCO gene percentage (positive). Moderate positive correlations were also observed between covered length and assembly length, gene number and N50 of contigs (Figure 4).

### 2.3. Efficiency Differences in Reference-Based Transcript Mapping in Rye

**Figure 5:**
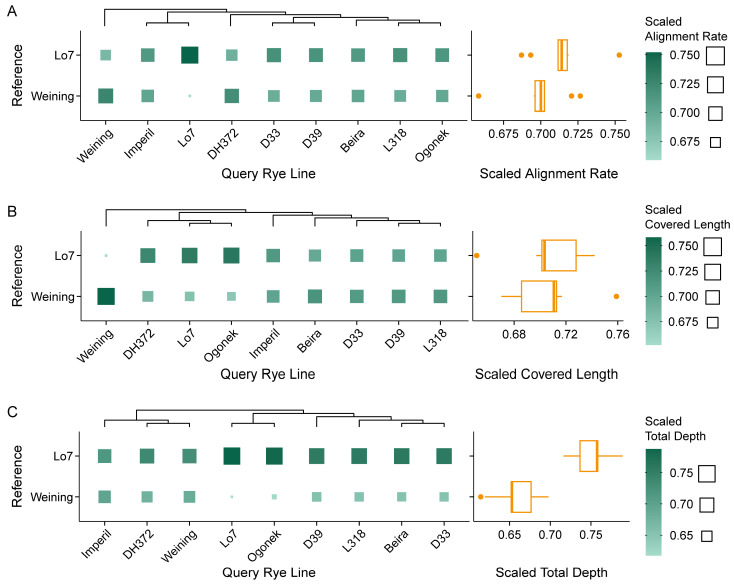
Distribution of alignment rate, covered length, and total depth of RNA-seq reads derived from different rye varieties mapped to different rye genome references. (**A**) Alignment rate. (**B**) Covered length. (**C**) Total depth. Scaled statistics are displayed via heatmap on the left and corresponding boxplot (statistics based on each reference) on the right. Original statistics are scaled according to each query rye line and ranked in descending order (e.g., Beira received the higher covered length when mapped to Weining but higher total depth when mapped to Lo7). The references are sorted according to the average scaled total statistics.

Mapping results of rye lines revealed that the alignment rate of Helltop was merely 22.8% on average. We suspect that Helltop was actually not a rye cultivar [45]. Therefore, Helltop was excluded from the subsequent analyses. The alignment rate of other rye lines ranged from 76.2% (Weining mapped to Lo7) to 96.1% (Imperil mapped to Lo7). The Lo7 assembly performed overall better than Weining in alignment rate, covered length, and total depth. However, mapping reads to the Weining genome assembly achieved larger covered length for Weining itself and rye lines Beira, D33, D39, and L318 (Figure 5). Similar to wheat, mapping of RNA reads from a line to its own assembly produced high yields.

### 2.4. Efficiency Differences in Reference-Based Transcript Mapping in Triticale

To date, there have been no triticale reference genome assemblies available. Therefore, we mapped the triticale cDNA reads (Table S1 and PRJCA035606 (https://ngdc.cncb.ac.cn/gsa, created in the present study)) to the genomes of their originating parents, namely wheat and rye. Results from single-parent references revealed that mapping to the reference assemblies of Kariega, Fielder, CWI86942, Chuanmai104, and Chinese Spring yielded higher alignment rates (Figure S2). The results of covered length were similar to those in wheat, with SY Mattis, Aimengniu, Shi4185, Kuofan11, and Xiaoyan6 ranking highest (Figure S3). For total depth, SY Mattis, Svevo, Zang1817, Zavitan, and Jagger showed the highest values (Figure S4). The single references of rye (Weining and Lo7) and allohexaploid wheat Renan performed poorly in terms of alignment rate, covered length, and total depth. Interestingly, although assemblies of the two allotetraploid wheat lines (Zavitan and Svevo) ranked high in total depth, they performed poorly in alignment rate and covered length.

**Figure 6:**
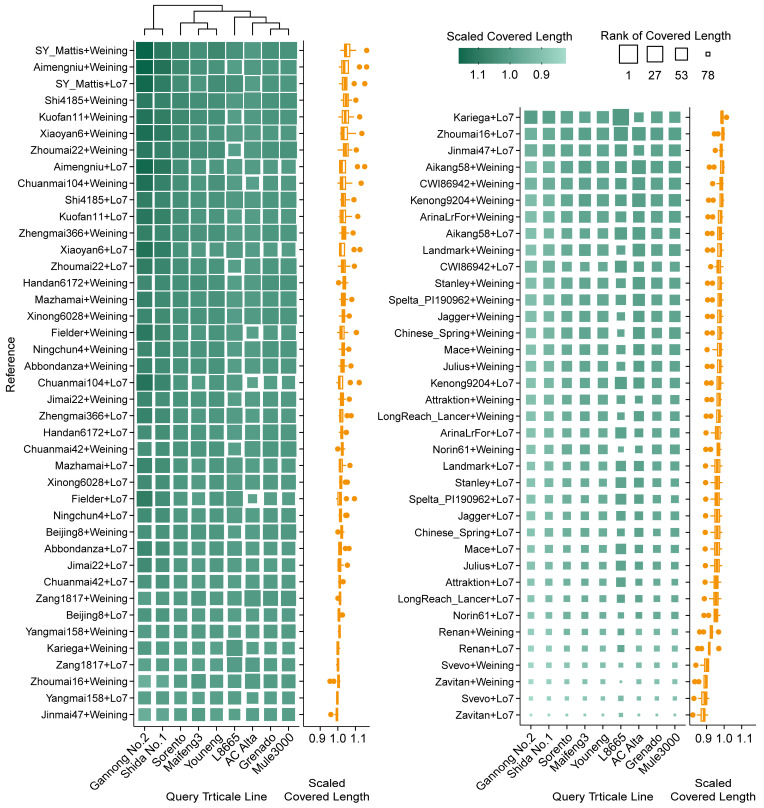
Covered length distribution of RNA-seq reads derived from different triticale varieties mapped to different simulated triticale genome references. Scaled covered lengths are displayed via heatmap on the left and corresponding boxplot (statistics based on each reference) on the right. Original covered lengths were scaled according to each query triticale line and ranked in descending order (e.g., AC Alta received the highest and lowest covered length when mapped to Shi4185 + Weining and Zavitan + Lo7, respectively). The references are sorted according to the average scaled covered length of all varieties.

**Figure 7:**
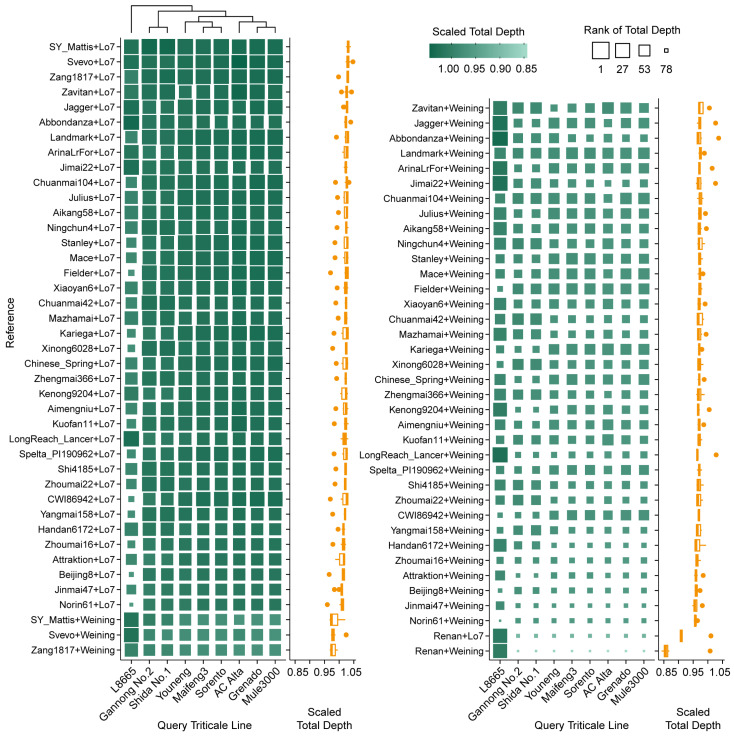
Total depth distribution of RNA-seq reads derived from different triticale varieties mapped to different simulated triticale genome references. Scaled total depths are displayed via heatmap on the left and corresponding boxplot (statistics based on each reference) on the right. Original total depths are scaled according to each query triticale line and ranked in descending order (e.g., AC Alta received the highest and lowest total depth when mapped to Svevo + Lo7 and Renan + Weining, respectively). The references are sorted according to the average scaled total depth of all varieties.

The typical triticale genome is a combination of wheat (either tetraploid or hexaploid) and rye. To interpret the real genome more closely, we combined wheat and rye assemblies and evaluated their performance in annotating RNA-seq data. The resulting alignment rate showed that the long-read L8665 dataset reached more than 99.9%. The alignment rates of other short-read datasets ranged from 88% to 93%, except for Shida No. 1 (~65%), evidencing the high reliability of the combined reference genome. Specifically, Kariega + Weining ranked first overall, followed by Kariega + Lo7, Zang1817 + Weining, ArinaLrFor + Weining, and Xinong6028 + Lo7 (Figure S5); the top combined covered lengths were achieved by SY Mattis + Weining, Aimengniu + Weining, SY Mattis + Lo7, Shi4185 + Weining, and Kuofan11 + Weining (Figure 6). The total depth was separated by the two rye assemblies into two main groups, with the top values all represented by combinations of Lo7 with SY Mattis, Svevo, Zang1817, Zavitan, and Jagger (Figure 7).

### 2.5. Karyotype Analysis Using RNA-Seq-Based Read Mapping in Triticale

**Figure 8:**
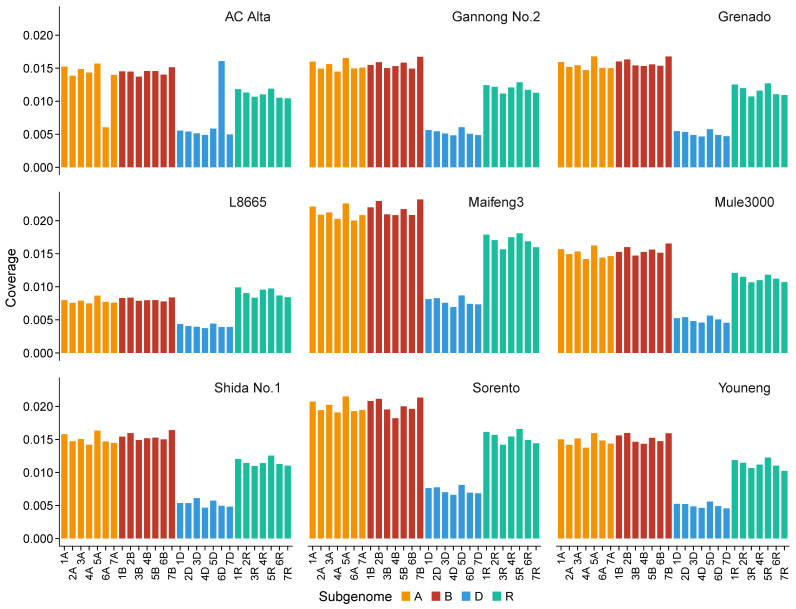
Chromosome-wise coverage distribution of RNA-seq reads derived from different triticale varieties mapped to genome reference of combined SY Mattis plus Lo7. Reads mapped to unassigned chromosomes are not displayed.

**Figure 9:**
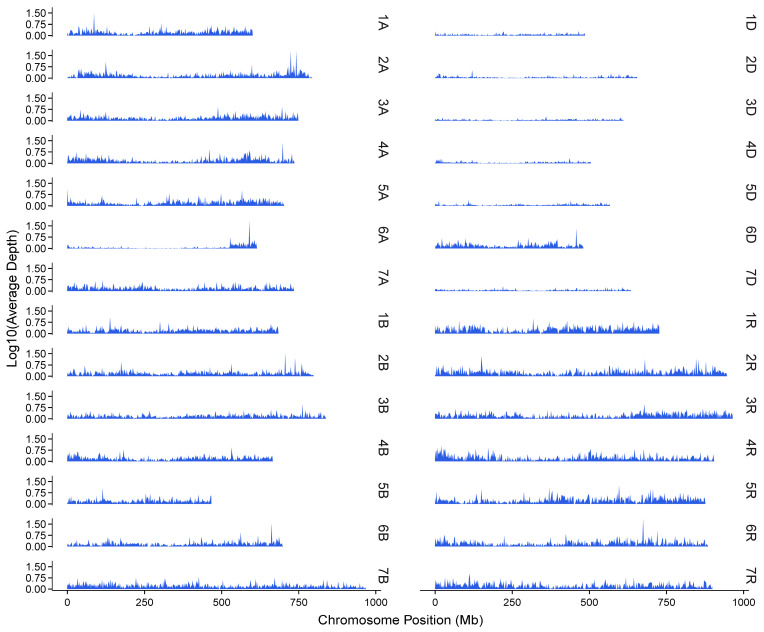
Read depth profile of RNA-seq reads derived from AC Alta mapped to genome reference of combined SY Mattis plus Lo7. Note the read depth distribution on chromosomes 6A and 6D. To ensure the y axis values were positive, average depths were increased by 1 before logarithmic transformation.

Hexaploid triticale is the most commonly cultivated form. However, chromosomal rearrangements, including replacement, addition, or loss of chromosome(s), as well as partial chromosomal translocations, have been observed in some genotypes [18]. Therefore, chromosome-wise distribution of reads was analyzed to identify the karyotype variations. Among the nine hexaploid triticale lines tested in the present study, read mapping of AC Alta displayed an abnormal decrease in coverage on chromosome 6A but an increase on 6D (Figure 8 and Figure S6), suggesting chromosome replacement or translocation events during the breeding of this line. A more detailed read distribution atlas indicated that stacks of reads were mapped to the entire chromosome 6D and only a short segment of 6A (Figure 9). Another example is Shida No. 1, in which a segment near the end of the 3D chromosome exhibited abnormal read aggregation, also resulting in increased read coverage and total depth of this chromosome (Figure 8 and Figures S6 and S7).

## 3. Discussion

A proper genome reference could improve the efficiency and accuracy of gene-related studies. By comparing the functional completeness and correctness of the published assemblies of wheat, rye, and triticale, we identified high-performance references that displayed universal applicability across different genotypes.

### 3.1. Correctness Rather than Number of Complete BUSCO Genes Is the Priority to Characterize Sequence Accuracy of Wheat Assemblies

BUSCO implements standardized benchmarking metrics for the presence of a predefined and expected set of single-copy marker genes as a proxy for genome-wide completeness [25]. BUSCO is particularly valuable for newly sequenced genomes and for comparing the completeness of different genome assemblies. In the present study, all the assemblies covered at least 96.75% of complete BUSCO genes, with completeness exceeding 99.37% in all wheat assemblies. The high percentage may be attributed to the large genome size of Triticeae species, and additionally the polyploid nature of wheat. Furthermore, polyploid genomes (e.g., hexaploid wheat) showed elevated proportions of duplicated BUSCO genes (hexaploid wheat vs. tetraploid wheat vs. rye). This pattern aligns with the inherent genomic redundancy of polyploid Triticeae species, where whole-genome duplication events amplify gene copy numbers. The overall trend was in line with the findings described previously [46].

Notably, differences in the percentages of complete BUSCO genes among wheat assemblies were minimal, and the scores displayed only moderate correlation with read mapping statistics from RNA-seq analysis (Figure 1 and Figure 4). In contrast, the difference in complete BUSCO genes between the two diploid rye lines was distinct (98.65% for Lo7 vs. 96.75% for Weining) and was in line with the RNA-seq validation metrics (Figure 1 and Figure 5). Similar results were also observed in other plants [47]. These observations question the efficacy of using BUSCO in assessing large, complex, and polyploid genome assemblies.

Base-level correctness of a genome assembly can be interpreted as the accuracy of nucleotide assignment, mainly including single-base substitutions and insertions/deletions. Evaluation of base-level correctness is often neglected, despite its essential role in genome quality [22]. Single-base errors generated during sequence assembly might introduce new start or stop codons, leading to false downstream annotations of coding sequences (CDS) and protein sequences, and disrupting BUSCO gene integrity. In our study, a significantly high negative correlation was observed between internal stop codon frequency in BUSCO genes and total depth in wheat (Figure 4). Considering the high conservation of BUSCO genes among different plant species, it is likely that the internal stop codons in BUSCO genes represent one type of base-level error during genome sequencing or assembly. Moreover, mapping depth has been reported as a direct indicator of the accuracy of RNA-seq data [29,30,31]. In the context of mapping various RNA-seq data to different genome assemblies, the variance in resulting mapping depth can also be evaluated to reflect the correctness of reference assemblies. Similarly, although just moderately correlated, the percentage of fragmented BUSCO genes serves as a reference for assessing long fragment and structural correctness of a genome assembly (Figure 4). Collectively, the internal stop codon frequency in BUSCO genes can be employed to evaluate the base-level correctness of existing and future genome assemblies.

### 3.2. Efficient References to Study Gene Structure and Expression in Wheat, Rye, and Triticale

Statistics of overall alignment rate, covered length, and depth generated via RNA-seq read mapping were employed to evaluate the transcript mappability of genome assemblies. Specifically, alignment rate represents the percentage of sequenced reads successfully mapped to a reference genome. Considering that some mapped reads are partially aligned to the genome or contain unmapped bases, alignment rate cannot faithfully reflect the absolute amount of mapped bases [48]. Therefore, total covered length and depth of mapped RNA-seq reads were preferentially selected to signify the completeness and correctness of the subject assemblies [28]. However, as a basic statistic of read mapping, alignment rate is easy to obtain and can be utilized to evaluate mapping quality. For example, in the current study, the alignment rate of a claimed rye cultivar Helltop was as low as 22.8% on average, which was evidently incorrect and should be excluded from downstream analyses to avoid wasting time and making erroneous decisions. In such cases, covered length and total depth cannot directly quantify the large number of unmapped bases.

Larger covered length represents a longer effective reference template in the assembly than other candidates, which is predominantly linked to the completeness of gene space. On the other hand, higher depth indicates that more reads were successfully aligned to the subject assembly than to other candidates, which implies a higher sequence similarity between the query line and the subject genome, and also ensures the accuracy of sequence structure and expression quantification [29,30,31]. Additionally, alignment rate in this study was regarded as an auxiliary metric for total depth evaluation.

For allohexaploid wheat, alignment to the assembly of SY Mattis achieved both the largest covered length and total depth on average across all query lines. SY Mattis is a European winter wheat variety that harbors numerous chromosome segment translocations [3,49,50]. Other top-ranked assemblies exhibited advantages specifically in either covered length or total depth. The widely adopted Chinese Spring assembly ranked in a lower position and was not recommended for gene-related studies unless using data from the same genotype. Nevertheless, a key advantage of the Chinese Spring assembly is its extensive gene annotations [32]. Researchers may need to map the existing annotations to genes in other assemblies, or select the Chinese Spring assembly directly if sequence completeness is not the priority. Similar considerations apply to triticale studies.

Although the assembly size of Lo7 is much smaller, and the predicted gene number is less than Weining, and was not recommended by Rabanus-Wallace et al. [51] due to the shorter scaffolds, the assembly of Lo7 ranked higher in all three metrics of covered length, total depth, and alignment rate on average (Table 1; Figure 5). The consistent results in rye and wheat indicated that the assembly continuity had limited influence on functional completeness and correctness, which is in line with the previously discovered poor correlation between BUSCO scores and N50 [47]. It should be noted that both of the two sequenced rye lines are self-pollinated, which is inconsistent with the general cultivated cross-pollinated cultivars [8,44]. Hence, relevant aspects should be taken into account when referring to these two assemblies. Moreover, the highly reticulate evolutionary history of rye is thought to have contributed to a highly mosaic genome structure, including frequent introgression and translocation of other Triticeae genome segments [44,52]. When analyzing structure or expression of rye genes, incorporation of other Triticeae genome resources may provide supplementary insights.

For hexaploid triticale, the top-performing assemblies can be summarized as a combination of top candidates of wheat and rye. Considering the large deficiency of total depth in the combined assembly of SY Mattis + Weining and Aimengniu + Weining, we recommend that the assembly of SY Mattis + Lo7 be prioritized as a mapping reference to balance mapping completeness and correctness (Figure 6 and Figure 7). As most cultivated triticale worldwide are hexaploid, covered length and depth designated on the D genome could result in a high probability of false positives. However, the octoploid reference is still recommended because introgression of the D genome into ABR genomes frequently occurs during triticale breeding, such as substitutions of 1D (1A), 2D (2R), 5D (5B) and 6D (6R), deletions of the long arm of chromosome 2D or the short arm of 5R, and translocations of the long arms of 7D/7A, the short arms of 6D/6A, or the long arms of 1D/1A [1,18,53]. Read mapping of AC Alta and Shida No. 1 in this study evidenced these modifications (Figure 9 and Figure S7). Moreover, Cao et al. [18] reported that among 199 accessions originally labeled as triticale, 20 were verified as hexaploid wheat, three as tetraploid wheat, and one as rye. An option to avoid or decrease artifacts is examining read distribution on chromosomes after read mapping to clarify potential mislabeling, chromosome substitutions, or large segment translocations, and excluding void mapping results on certain chromosomes (usually D genomes) in downstream analyses. However, reads mapped to sequences on unassigned chromosomes are difficult to identify or remove and require further consideration.

## 4. Materials and Methods

### 4.1. Data Source

Genome sequences and corresponding annotations of wheat and rye, publicly available RNA-seq data for wheat and rye, and triticale cultivars AC Alta, Gannong No. 2, Shida No. 1, and L8665 were downloaded from the relevant repositories described in each reference publication. The basic information and sources of the data are listed in Table 1 and Table S1. Transcriptomic data of triticale cultivars Youneng, Maifeng3, Sorento, Grenado, and Mule3000 were obtained through local RNA-seq analysis as described in subsequent Section 4.3 and Section 4.4 below.

### 4.2. BUSCO Assessing of Genome Assemblies

Completeness of genome assemblies was assessed using BUSCO (version 5.7.1). Genes were mapped via the Miniprot pipeline and the poales_odb10 gene set was used as the reference. Statistics of the assemblies and annotations were summarized using BUSCO and assembly_stats (version 0.1.4).

### 4.3. Plant Materials and Growth Conditions

The triticale used for seedling growth and RNA extraction in this study included cultivars Youneng, Maifeng3, Sorento, Grenado, and Mule3000. Healthy and uniform seeds were surface-sterilized by soaking in 10% hydrogen peroxide for 30 min, followed by rinsing in distilled water five times. The seeds were then placed on filter paper in 15 cm diameter petri dishes and kept in a growth chamber to select uniformly germinated seeds. After one day, seeds with protruding radicles were carefully transplanted to rectangular pots (10 cm × 10 cm × 8.5 cm) with perlite as the culture substrates. Deionized water and 1/2-strength Hoagland solution were applied alternately every other week. Throughout cultivation, the growth chamber was maintained at a photoperiod of 16 h light/8 h dark and a temperature of 25/20 °C (light/dark). After three weeks, the shoots were collected, immediately frozen in liquid nitrogen, and stored at −80 °C until RNA extraction.

### 4.4. RNA Extraction, RNA-Seq Library Construction and Transcriptome Sequencing

Total RNA of triticale cultivars Youneng, Maifeng3, Sorento, Grenado, and Mule3000 was extracted using RNAprep Pure Plant Kit (Tiangen Biotech, Beijing, China) according to the manufacturer’s instructions. After concentration and quality examination, mRNA was purified from total RNA using oligo(dT)-attached magnetic beads for transcriptome sequencing. The subsequent RNA fragmentation, cDNA synthesis, sequencing library construction, and qualification processes were performed using the NEBNext Ultra RNA Library Prep Kit for Illumina (NEB, Ipswich, MA, USA) according to the manufacturer’s instructions. The qualified libraries were pooled and sequenced using a paired-end 150 bp (PE150) strategy on the Illumina NovaSeq 6000 platform at Novogene Bioinformatics Technology Co., Ltd. (Beijing, China). The generated sequences were deposited in the China National Center for Bioinformation under project number PRJCA035606 and are publicly accessible at https://ngdc.cncb.ac.cn/gsa (date created: 26 January 2025).

### 4.5. RNA-Seq Data Analysis

All sequence data generated via Next-Generation Sequencing, including those obtained from public repositories and those generated in this study (Table S1), were processed in the same downstream pipeline. Sequence data from the same crop variety and experiment were merged prior to processing. Quality control of raw sequence data was performed using fastp (version 0.23.2) to remove reads containing adapters or of low quality. The main filtering criteria included qualified quality phred (5), unqualified percent limit (50), limit of number of N base (15), and minimal required read length (60% of total). The maximum number and percentage of mismatched bases to detect overlapping regions of PE reads were set to 1 and 10, respectively. After building indices based on the corresponding GTF annotation for high-confidence (HC) genes, the obtained clean reads from each genotype were aligned separately to the reference genomes using hisat2 (version 2.2.1) with the default settings. The alignment rate of query RNA-seq reads was calculated from hisat2 outputs. After mapping, the SAM files generated by hisat2 were sorted and converted to BAM format using Samtools (version 1.16.1) before downstream analyses.

Sequence data of triticale cultivar L8665 were generated using Oxford Nanopore MinION platform [54] and were processed differently. Briefly, raw sequences were filtered using Filtlong (version 0.2.1) with minimum length threshold and minimum mean quality threshold set to 500 and 7, respectively. The resulting clean reads were then mapped to reference genomes via minimap2 (version 2.28), and alignment metadata were retrieved using Samtools flagstat, followed by calculating via a local Perl script.

Based on the BAM files generated by sequence alignment, mapping coverage and depth of mapped reads were analyzed using PanDepth (version 2.25) and further summarized and visualized in R.

## 5. Conclusions

In this study, assemblies of wheat, rye, and triticale were analyzed through integrated BUSCO analysis and transcript mapping to select high-performance references for studies of gene space and expression. Comparison of alignment rate, covered length, and total depth for RNA-seq read mapping revealed that the assemblies of SY Mattis, Lo7, and SY Mattis plus Lo7 were the most robust references for gene-related studies in wheat, rye, and triticale, respectively. Most BUSCO statistics for wheat genomes were not sensitive enough to evaluate the genome assemblies, but the frequency of internal stop codons in BUSCO genes was closely correlated with RNA-seq read mapping depth, which was considered to be an indicator of sequence correctness of a genome assembly. For hexaploid triticale, the combination of AABBDDRR genomes provides a comprehensive reference that included the frequently incorporated D genome sequences in the genome. Reference assemblies recommended in this study could assist future gene-related research, and the BUSCO indicators could serve to evaluate plant genome assemblies in the future.

## Data Availability

The raw sequence data reported in this paper have been deposited in the Genome Sequence Archive (Genomics, Proteomics & Bioinformatics 2021) in the National Genomics Data Center, China National Center for Bioinformation/Beijing Institute of Genomics, Chinese Academy of Sciences (PRJCA035606), and are publicly accessible at https://ngdc.cncb.ac.cn/gsa (date created: 26 January 2025).

## Supplementary Materials

The following supporting information can be downloaded at https://www.mdpi.com/article/10.3390/plants14071140/s1. Figure S1: Alignment rate distribution of RNA-seq reads derived from different wheat varieties mapped to different wheat genome references. Figure S2: Alignment rate distribution of RNA-seq reads derived from different triticale varieties mapped to different wheat or rye genome references. Figure S3: Covered length distribution of RNA-seq reads derived from different triticale varieties mapped to different wheat or rye genome references. Figure S4: Total depth distribution of RNA-seq reads derived from different triticale varieties mapped to different wheat or rye genome references. Figure S5: Alignment rate distribution of RNA-seq reads derived from different triticale varieties mapped to different simulated triticale genome references. Figure S6: Chromosome-wise total depth distribution of RNA-seq reads derived from different triticale varieties mapped to genome reference of combined SY Mattis plus Lo7. Figure S7: Read depth profile of RNA-seq reads derived from Shida 1 mapped to genome reference of combined SY Mattis plus Lo7. Table S1: Information on RNA-seq data used in this study.
