# Supplementary material for: Evaluating Genome Assemblies for Optimized Completeness and Accuracy of Reference Gene Sequences in Wheat, Rye, and Triticale
Source: Plants (Basel). 2025 Apr 6;14(7):1140. doi: 10.3390/plants14071140 (PMC11991537; doi:10.3390/plants14071140)
Supplement: Supplementary file 1 [file plants-14-01140-s001.zip › Supplementary Figures.pdf]

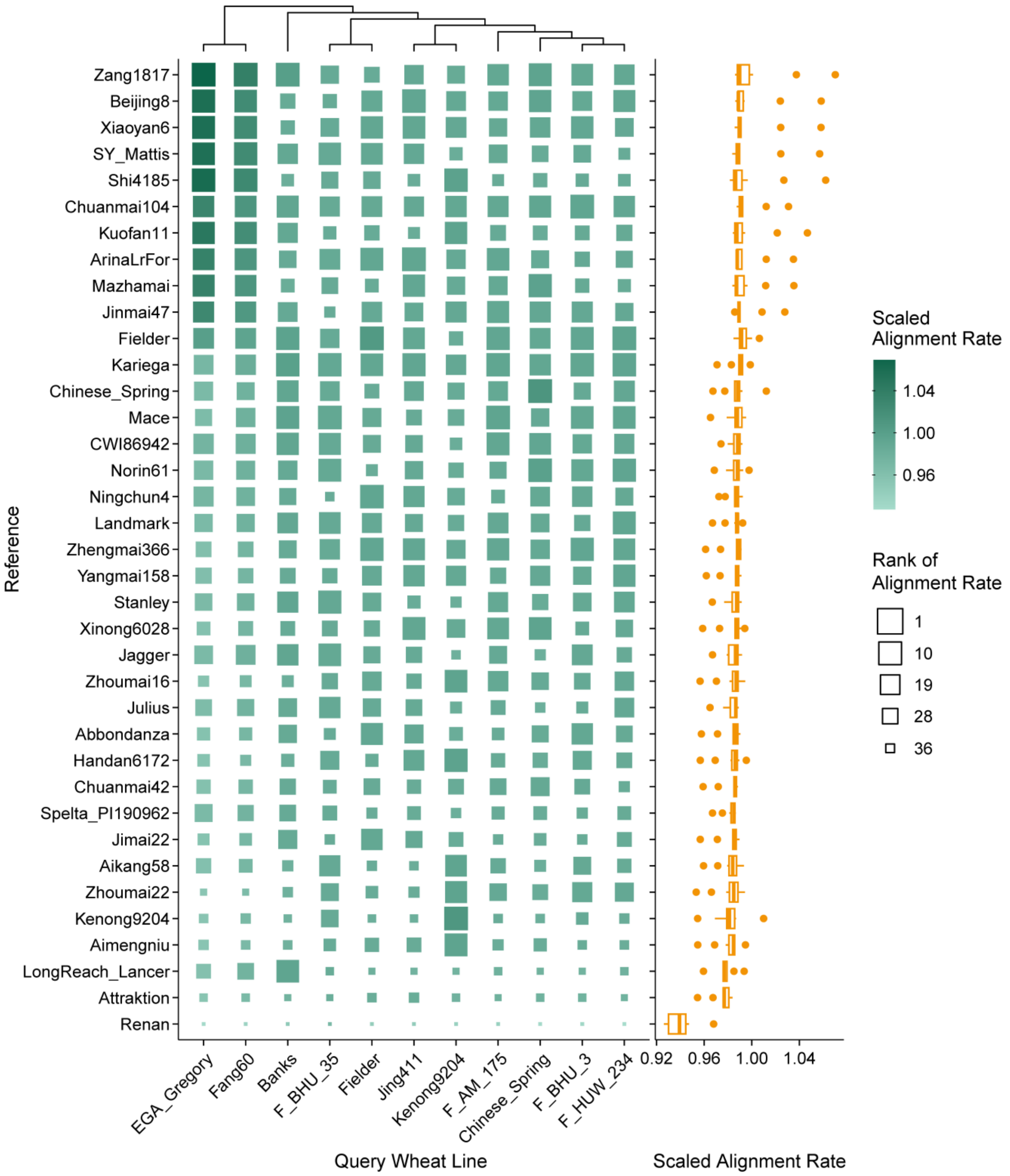

Figure S1. Alignment rate distribution of RNA-Seq reads derived from different wheat varieties mapped to different wheat genome references. Scaled alignment rates were displayed via heatmap on the left and corresponding boxplot (statistics based on each reference) on the right. Original alignment rates were scaled according to each query wheat line and ranked in descending order. The references were sorted according to the average scaled alignment rate of all varieties.

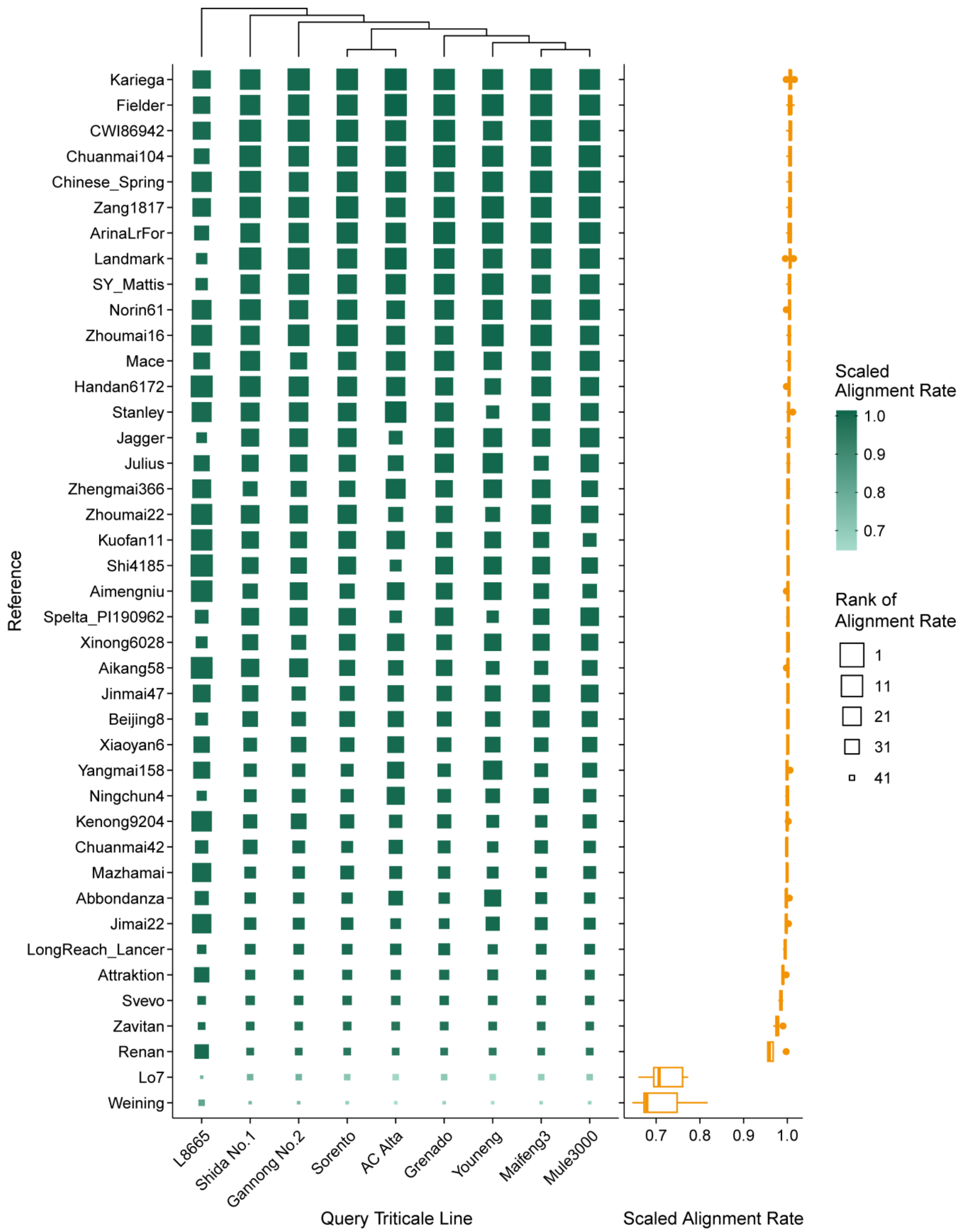

Figure S2. Alignment rate distribution of RNA-Seq reads derived from different triticale varieties mapped to different wheat or rye genome references. Scaled alignment rates were displayed via heatmap on the left and corresponding boxplot (statistics based on each reference) on the right. Original alignment rates were scaled according to each query triticale line and ranked in descending order. The references were sorted according to the average scaled alignment rate of all varieties.

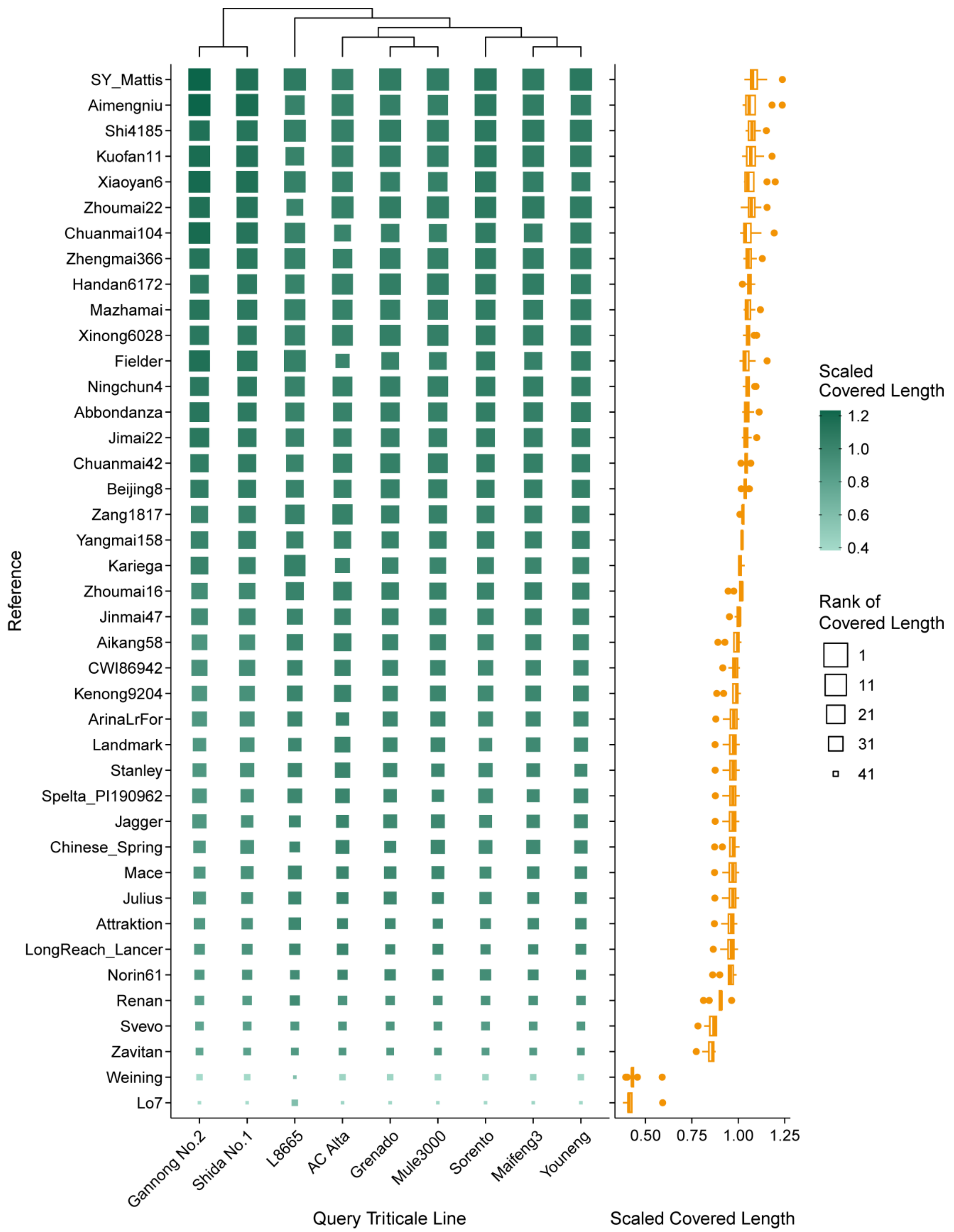

Figure S3. Covered length distribution of RNA-Seq reads derived from different triticale varieties mapped to different wheat or rye genome references. Scaled covered lengths were displayed via heatmap on the left and corresponding boxplot (statistics based on each reference) on the right. Original covered lengths were scaled according to each query triticale line and ranked in descending order. The references were sorted according to the average scaled covered length of all varieties.

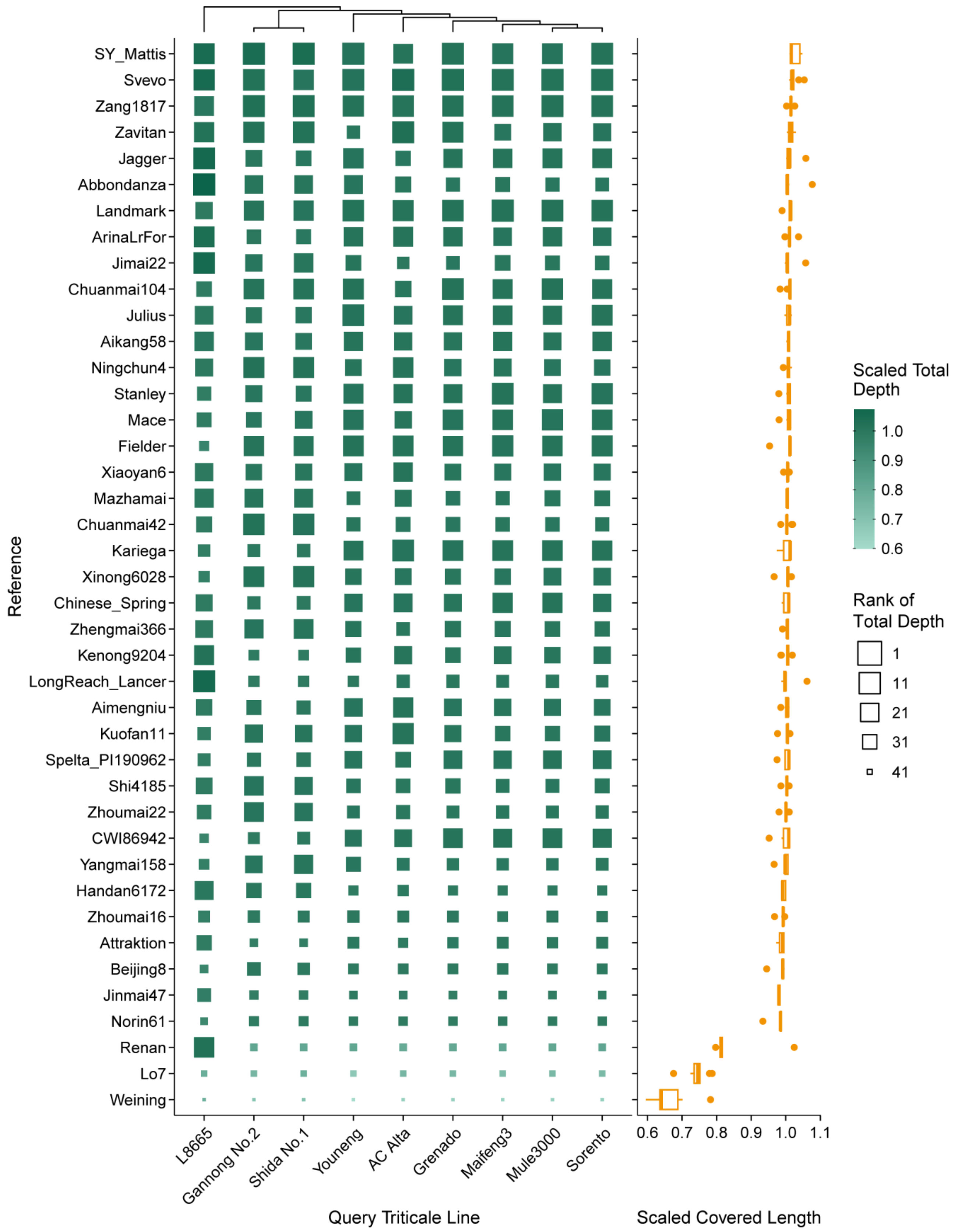

Figure S4. Total depth distribution of RNA-Seq reads derived from different triticale varieties mapped to different wheat or rye genome references. Scaled total depths were displayed via heatmap on the left and corresponding boxplot (statistics based on each reference) on the right. Original total depths were scaled according to each query triticale line and ranked in descending order. The references were sorted according to the average scaled total depth of all varieties.

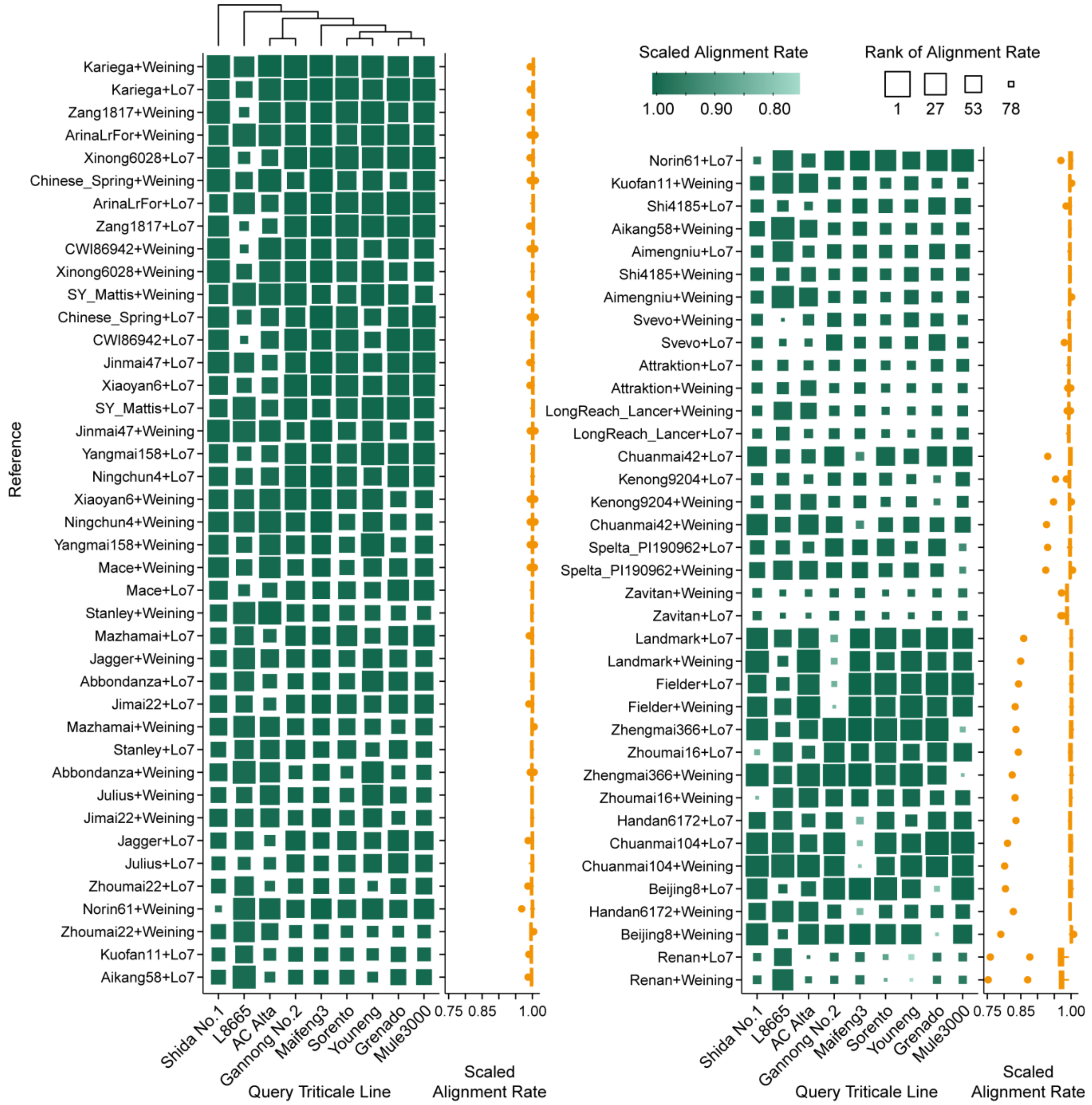

Figure S5. Alignment rate distribution of RNA-Seq reads derived from different triticale varieties mapped to different simulated triticale genome references. Scaled alignment rates were displayed via heatmap on the left and corresponding boxplot (statis-tics based on each reference) on the right. Original alignment rates were scaled according to each query triticale line and ranked in descending order. The references were sorted according to the average scaled alignment rate of all varieties.

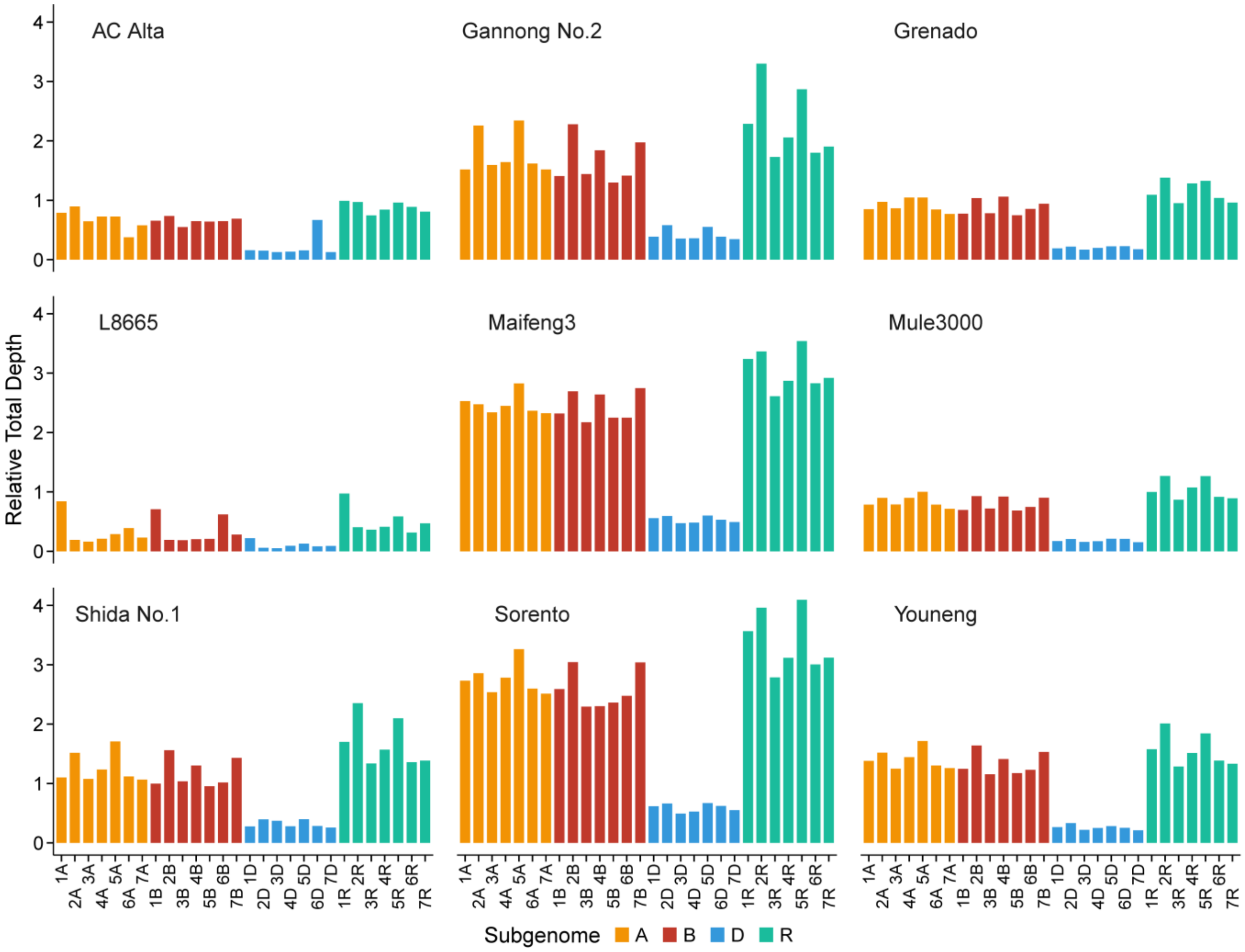

Figure S6. Chromosome-wise total depth distribution of RNA-Seq reads derived from different triticale varieties mapped to genome reference of combined SY Mattis plus Lo7. Reads mapped on unassigned chromosomes were not displayed. Relative total depth was calculated as original total depth divided by the length of each chromosome.

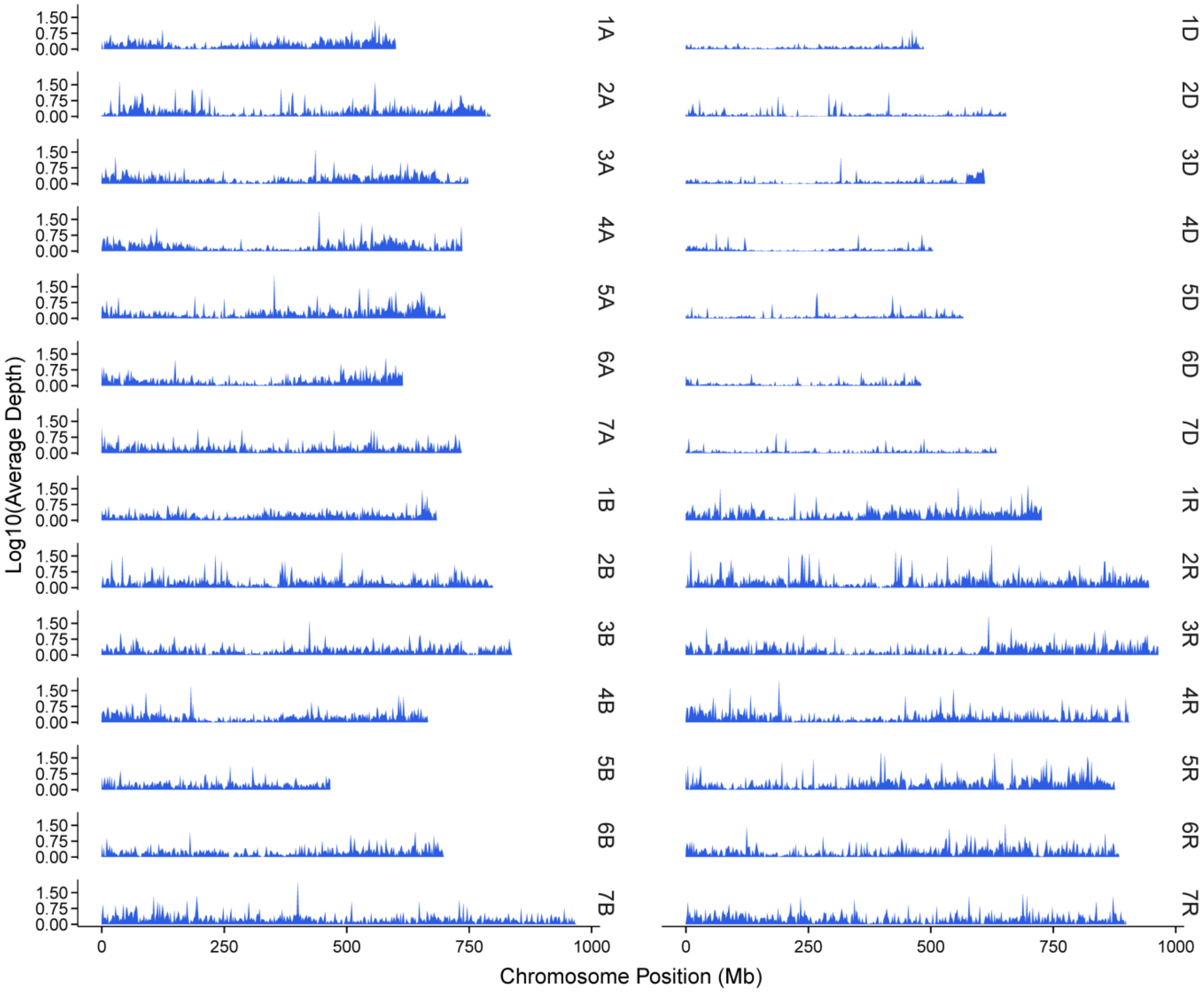

Figure S7. Read depth profile of RNA-Seq reads derived from Shida No. 1 mapped to genome reference of combined SY Mattis plus Lo7. To ensure the y axis value were positive, average depth were added by 1 before logarithmic transformation.
